# Single-organ pulmonary metastasis is a favorable prognostic factor in metastatic colorectal cancer patients treated with FOLFIRI and vascular endothelial growth factor inhibitors

**DOI:** 10.1186/s12885-023-11067-y

**Published:** 2023-07-06

**Authors:** Koshiro Fukuda, Hiroki Osumi, Koichiro Yoshino, Izuma Nakayama, Shota Fukuoka, Mariko Ogura, Takeru Wakatsuki, Akira Ooki, Daisuke Takahari, Keisho Chin, Kensei Yamaguchi, Eiji Shinozaki

**Affiliations:** 1 Department of Gastroenterology, Cancer Institute Hospital, Japanese Foundation for Cancer Research, Tokyo, Japan; 2 Department of Gastroenterology, Graduate School of Medicine, The University of Tokyo, Tokyo, Japan

**Keywords:** FOLFIRI, Metastatic colorectal cancer, Single organ pulmonary metastasis, Vascular endothelial growth factor inhibitor, Prognosis, Progression-free survival, Overall survival

## Abstract

**Background:**

Few studies have focused on the impact of single-organ pulmonary metastases on progression-free survival and overall survival in patients with metastatic colorectal cancer. Recognizing differences in prognosis and chemotherapeutic efficacy based on metastasized organs may help in optimizing treatment strategies. The exploratory study was conducted to evaluate the comparative clinical outcomes and prognoses of patients with metastatic colorectal cancer presenting with single-organ pulmonary metastases and treated with folinic acid, 5-fluorouracil, irinotecan, and vascular endothelial growth factor inhibitors as second-line chemotherapy.

**Methods:**

This retrospective study included 289 patients with metastatic colorectal cancer treated with second-line folinic acid, 5-fluorouracil, irinotecan, and vascular endothelial growth factor inhibitors. The response rate, disease control rate, progression-free survival, and overall survival were assessed in the participants.

**Results:**

Among the 289 patients enrolled, 26 (9.0%) had single-organ pulmonary metastasis with left-sided primary locations, lower levels of tumor markers at the initiation point of chemotherapy, a significantly higher disease control rate (96.2% vs. 76.7%, *P* = .02), and a longer progression-free survival (median 29.6 months vs. 6.1 months, *P* < .001) and overall survival (median 41.1 months vs. 18.7 months, *P* < .001) than patients with other forms of metastatic colorectal cancer. Multivariate analysis showed that single-organ pulmonary metastasis was an independent predictor of longer progression-free survival (hazard ratio 0.35, *P* = .00075) and overall survival (hazard ratio 0.2, *P* = .006).

**Conclusion:**

Single-organ pulmonary metastasis was a strong predictor of progression-free survival and overall survival in patients with metastatic colorectal cancer treated with folinic acid, 5-fluorouracil, irinotecan, and vascular endothelial growth factor inhibitors as second-line chemotherapy; this provides preliminary evidence for medical guidelines and clinical decision-making on novel therapeutic strategies for these patients.

## Background

Colorectal cancer (CRC) is the fourth most fatal cancer worldwide, with approximately 900,000 deaths annually. Moreover, this cancer accounts for approximately 10% of all cancers diagnosed and cancer-related deaths worldwide each year [1]. It is the second most common cancer in women and the third most common cancer in men [2]. Among those diagnosed with CRC, 20% have metastatic colorectal cancer (mCRC), and 40% of patients with localized cancer present with recurrence following previous treatment [3]. Although new treatment strategies (including molecularly-targeted drugs) have been developed and the prognosis of CRC has consequently improved, the prognosis for metastatic CRC remains poor, with a five-year survival rate of less than 20% [4].

The lungs are one of the most frequent metastatic sites arising from mCRC, with an incidence of approximately 10–25% [5–7]. The most common primary tumors to result in pulmonary metastases include breast cancer, CRC, renal carcinoma, uterine leiomyosarcoma, and head and neck carcinoma [8]. Compared to colon cancer, patients with rectal cancer are at higher risk of synchronous and metachronous pulmonary metastases [9–11] due to the direct spread of rectal cancer cells into the systemic circulation through the hemorrhoidal veins [12]. In a multivariate analysis of 5,673 Stage I–IV CRC patients, those with single-organ pulmonary metastases showed a significantly better prognosis compared to those with metastasis at a different location in addition to lung metastasis [10]. According to another retrospective study, patients with single-organ pulmonary lesions have a significantly better prognosis as compared to those with a single-organ metastasis to another organ [13].

Surgical resection has gradually been accepted as an appropriate treatment for single-organ pulmonary metastases arising from mCRC [5, 7, 14]. In Japan, indications for pulmonary metastasectomy follow the criteria outlined in the Japanese Society for Cancer of the Colon and Rectum (JSCCR) guidelines [15] which are as follows: (1) the patient is capable of tolerating surgery; (2) the primary colorectal tumor is controlled or can be controlled; (3) the metastatic lung tumor can be completely resected; (4) any extra-thoracic metastases can be controlled; and (5) the function of the remaining lung would be adequate after metastasectomy.

Although the efficacy of pulmonary resection for solitary pulmonary metastases has been established, pulmonary resection for multiple or bilateral lesions remains controversial. Recently, several studies have reported on novel treatment strategies, including stereotactic radiation therapy (SRT) and radiofrequency ablation (RFA), for addressing pulmonary metastases arising from mCRC [16, 17]. However, these strategies have not yet been established. Patients with multiple metastatic pulmonary lesions are treated with chemotherapy as these multiple lesions are thought to represent systemic dissemination and are unresectable [2]. Although there have been several reports related to the treatment of single-organ liver metastasis of mCRC [18–20], few studies are related to the treatment of single-organ pulmonary metastasis of mCRC. Therefore, it is unclear wheather single-organ metastases to the lung can affect the outcomes of patients with mCRC treated with chemotherapy.

Angiogenesis is closely related to pulmonary metastasis in some cancers. Ghouse et al. found that angiogenic switching in the lungs prior to the arrival of tumor cells in a mouse model of breast carcinoma and the subsequent angiogenesis contributes to the premetastatic niche in rapidly progressing cancers and that inhibiting this process is beneficial for reducing pulmonary metastases [21]. Clinical trials have shown that vascular endothelial growth factor (VEGF) inhibitors in combination with folinic acid, 5-fluorouracil, and irinotecan (FOLFIRI) significantly improved overall survival (OS) as compared with FOLFIRI alone [22–24]; therefore, these combination therapies are a standard second-line treatment.

We conducted the exploratory study to evaluate the comparative clinical outcomes and prognoses of patients with mCRC presenting with single-organ pulmonary metastases and treated with FOLFIRI and VEGF inhibitors as second-line chemotherapy.

## Methods

### Study population

This retrospective study included 289 patients with mCRC who were treated with FOLFIRI and VEGF inhibitors as a second-line chemotherapy regimen from January 2017 to December 2019 at our institution. The study cohort included patients with mCRC at the lungs, livers, lymph nodes, and peritoneum as well as other locations and patients with mCRC who consistently had only pulmonary metastasis from the time of initial diagnosis until the start of the second line were considered mCRC patients with single-organ pulmonary metastasis. For the analysis, we investigated the association between single-organ pulmonary metastases and treatment efficacy and prognosis in patients treated with FOLFIRI and VEGF inhibitors as second-line chemotherapy.

This study was approved by the institutional review board of the Cancer Institute Hospital of Japanese Foundation of Cancer Research (IRB receipt number: 2020-GA-1017) and was conducted in accordance with the principles of the 1964 Declaration of Helsinki. The study protocol was described on the hospital website, and the patients were provided the opportunity to opt out. Therefore, no additional consent was required from the enrolled patients.

### Treatment schedule

Bevacizumab (BEV) was administered at the recommended dose of 5 mg/kg, ramucirumab (RAM) at the recommended dose of 8 mg/kg, and aflibercept (AFL) at the recommended dose of 4 mg/kg. The concomitant chemotherapy was FOLFIRI (irinotecan 150–180 mg/m^2^, L-leucovorin 200 mg/m^2^, bolus 5-FU 400 mg/m^2^, 46-h infusion of 5-FU 2,400 mg/m^2^). Prophylactic treatments and dose reductions were performed based on the recommendations delineated within the established guidelines and the physician’s judgment.

### Assessments

Patient data were collected from medical records and imaging scans (enhanced or plain Computed Tomography (CT)) for age, sex, primary tumor location (right-sided [cecum, ascending colon, or transverse colon] or left-sided colon [descending colon, sigmoid colon, or rectum]), metastatic site, *RAS* status in the tissue, prior BEV exposure in first-line chemotherapy, first-line progression-free survival (PFS; in patients treated with BEV only), relapse within 6 months of completing oxaliplatin-based adjuvant therapy, and serum markers (carcinoembryonic antigen [CEA], carbohydrate antigen 19–9 [CA19-9] and lactate dehydrogenase [LDH]).

Complete response (CR), partial response (PR), stable disease (SD), and progressive disease (PD) were defined based on the RECIST guidelines (v1.1) [25]. The response rate (RR) denotes the proportion of patients experiencing a CR or PR to second-line chemotherapy, and the disease control rate (DCR) indicates the proportion of patients experiencing a CR, PR, or SD response to chemotherapy. PFS was defined as the time elapsing from the first day of second-line treatment to either the first objective evidence of disease progression or death from any cause; and OS was the time elapsing from the first day of second-line treatment to death. The grade of adverse events (AEs) was assessed using the Common Toxicity Criteria for Adverse Events (CTCAE) v5.0 [26].

### Statistical analyses

The primary endpoints of this study were PFS and OS from the time of induction of FOLFIRI and VEGF inhibitors as second-line chemotherapy. Data are reported as medians (ranges), means (ranges), or counts (percentages), as appropriate. Categorical variables were compared using Fisher’s exact tests, while continuous variables were compared using two-sample t-tests. Time-to-event analyses using Kaplan–Meier curves were performed to compare groups using log-rank tests. Multivariable Cox proportional hazard regression was used to adjust for possible confounders, including sex, age, primary tumor location, the presence of lung metastases only, tissue *RAS* mutations, prior BEV exposure in first-line chemotherapy, and treatment regimen.

All statistical tests were two-sided, and the significance was set at *P* < 0.05. The statistical analyses were performed using EZR statistical software (Saitama Medical Center, Jichi Medical University, Saitama, Japan), a graphical user interface for R (The R Foundation for Statistical Computing, Vienna, Austria). More precisely, EZR is a modified version of the R commander designed to add specific statistical functions frequently used in biostatistics [27].

## Results

### Cohort characteristics

In total, 289 patients were treated with second-line FOLFIRI and VEGF inhibitors (BEV, RAM, and AFL), of whom 137 (47.4%) were males and 152 (52.6%) were females, with a median age of 63 (range: 31–84) years; 26 patients (9.0%) had single-organ pulmonary metastasis. The liver was the most frequent site of metastasis (51.2% [148/289]), followed by the lungs (50.2% [145/289]), lymph nodes (34.3% [99/289]), and peritoneum (33.6% [97/289]). Eastern Cooperative Oncology Group Performance Status (ECOG PS) 0/1/2 was 232(80.3%) /56(19.4) /1(0.3%), respectively. Patients with single-organ pulmonary metastasis had significantly better PS than others (*P* = 0.036).

The study included 22 patients (84.6%) with pulmonary lesions on both sides and 4 patients (15.4%) with unilateral lesions. The median number of pulmonary lesions was 4.5 (1–53) lesions with a median maximum diameter of 12.5 mm (5–33) mm. Second-line treatment regimens were as follows: 119 patients (41.2%) received FOLFIRI and BEV, 107 patients (37.0%) underwent FOLFIRI and RAM, and 63 patients(21.8%) underwent FOLFIRI and AFL regimens. There were no significant differences in age, sex, or type of VEGF inhibitor between the study groups. None of the patients underwent any surgeries and locoregional therapies for metastases just before and after second line chemotherapy. This cohort also included 41 patients (14.2%) treated with anti-epidermal growth factor receptor (EGFR) monoclonal antibodies as first line treatment.

Of the 289 patients, 93 (32.2%) had right-sided colon tumors and 196 (67.8%) had left-sided tumors. The primary tumor sites were right-sided in 4 (15.4%) and left-sided in 22 (84.6%) patients with single-organ pulmonary metastases (*P* = 0.08).

In terms of *RAS/BRAF* status in the tissue, 132 (45.7%) of the tumors were wild-type, 151 (52.2%) showed mutations, and 6 (2.1%) had an unknown status. *RAS* variants are shown in Table 1. In cases with single-organ pulmonary metastases, *RAS* status in the tissue was wild-type in 12 patients (46.2%) and mutant in 14 patients (53.8%). The identified *RAS* variants were as follows*: KRAS* G12D (five patients [19.2%, *P* > 0.99]), *KRAS* G13D (three patients [11.5%, *P* = 0.72]), *KRAS* G12V (two patients [7.7%, *P* > 0.99]), *KRAS* G12C (two patients [7.7%, *P* = 0.16]), and *NRAS* Q61 (two patients [7.7%, *P* = 0.13]). The rates of *KRAS* G12C and *NRAS* Q61H mutations tended to be high in patients with single-organ pulmonary metastases, whereas none of the patients with single-organ pulmonary metastases had a *BRAF* V600E mutation. It was noted that 159 patients (55%) underwent prior BEV treatment in first-line chemotherapy.

**Table 1:** Patient demographic and clinical characteristics

Characteristics	All patients (*N* = 289) N (%)	Lung metastasis only (*N* = 26) N (%)	Others (*N* = 263) N (%)	*P* value
Age at enrollment (years)				
Median (range)	63.0 (31.0–84.0)	60.0 (38.0–80.0)	63.5 (31.0–84.0)	.12
Sex				
Male	137 (47.4)	16 (61.5)	121 (46.0)	.15
Female	152 (52.6)	10 (38.5)	142 (54.0)	
ECOG PS				
0	232	25	207	.036
1	56	1	55	
2	1	0	1	
Treatment regimen				
FOLFIRI + bevacizumab	119 (41.2)	14 (53.8)	105 (39.9)	.27
FOLFIRI + ramucirumab	107 (37.0)	6 (23.1)	101 (38.4)	
FOLFIRI + aflibercept	63 (21.8)	6 (23.1)	57 (21.7)	
Primary site				
Right-sided colon	93 (32.2)	4 (15.4)	89 (33.8)	.08
Left-sided colon	196 (67.8)	22 (84.6)	174 (66.2)	
*RAS/BRAF* status				
Wild-type	132 (45.7)	12 (46.2)	120 (45.6)	> .99
Mutant	151 (52.2)	14 (53.8)	137 (52.1)	
*KRAS* G12D	53 (18.3)	5 (19.2)	48 (18.3)	> .99
*KRAS* G13D	26 (9.0)	3 (11.5)	23 (8.7)	.72
*KRAS* G12V	27 (9.3)	2 (7.7)	25 (9.5)	> .99
*KRAS* G12C	8 (2.8)	2 (7.7)	6 (2.3)	.16
*NRAS* Q61	7 (2.4)	2 (7.7)	5 (1.9)	.13
*BRAF* V600E	8 (2.8)	0 (0)	8 (3.0)	> .99
Others unknown	6 (2.1)	0 (0)	6 (2.3)	
Prior bevacizumab exposure (in first-line chemotherapy)				
Yes	159 (55.0)	12 (46.2)	147 (55.9)	.41
No	130 (45.0)	14 (53.8)	116 (44.1)	
Serum markers (at initiation of second-line chemotherapy)				
CEA, median (range)	17.3 (0.5–17,056)	3.1 (1.2–466)	20.9 (0.5–17,056)	.004
CA19-9, median (range)	32.7 (2.0–50,000)	8.9 (2.0–1,293)	35.9 (2.0–50,000)	< .001
LDH, mean (range)	306 (119–5,753)	204 (119–307)	316 (134–5,753)	.18

*CA19-9* Carbohydrate antigen 19–9, *CEA* Carcinoembryonic antigen, *ECOG PS* Eastern Cooperative Oncology Group Performance status, *FOLFIRI*, Leucovorin + fluorouracil + irinotecan, *LDH*, Lactate dehydrogenase, *RAS* Rat sarcoma viral oncogene homolog

The median CEA of the patients was 17.3 (range: 0.5–17,056.1) ng/mL and the median CA19-9 was 32.7 (2.0–50,000) U/mL. The evaluated tumor markers (CEA and CA19-9) were significantly lower at the time of initiation of chemotherapy (CEA, *P* = 0.004; CA19-9, *P* < 0.001) in patients with single-organ pulmonary metastases than tumor markers in those with other forms of mCRC. In addition, the mean LDH level at the start of second-line chemotherapy treatment was 204 (119–307) U/L in patients with single-organ pulmonary metastases, while the mean LDH level was 316 (134–5,753) U/L in patients without these metastases (*P* = 0.18). The mean LDH level of patients with single-organ pulmonary metastases tended to be lower than that of patients with other forms of mCRC.

### Clinical outcomes

To assess the efficacy of FOLFIRI and VEGF inhibitors in patients with mCRC presenting with single-organ pulmonary metastases, we compared the clinical outcomes between mCRC patients with or without single-organ pulmonary metastases. The response rate (RR) for second-line FOLFIRI and VEGF inhibitors was 23.1% (6/26) in mCRC patients with single-organ pulmonary metastases and 16.1% (38/236) in patients with other forms of mCRC (*P* = 0.41). The DCR was 96.2% (25/26) in patients with single-organ pulmonary metastases and 76.7% (181/236) in patients with other forms of mCRC. (*P* = 0.02).

**Fig. 1:**
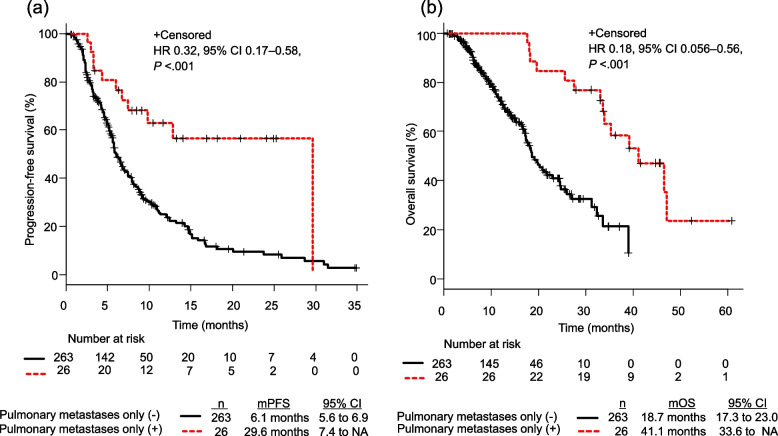
PFS and OS of patients with mCRC treated with FOLFIRI and anti-angiogenic drugs. **a** PFS and **b** OS in patients with single organ pulmonary metastases arising from mCRC after second-line chemotherapy as compared to those with other metastatic lesions. CI, confidence interval; FOLFIRI, leucovorin + fluorouracil + irinotecan; mCRC, metastatic colorectal cancer; mOS, months of overall survival; mPFS, months of progression-free survival; OS, overall survival; PFS, progression-free survival

The median PFS in patients with single-organ pulmonary metastases was 29.6 months, whereas that for other mCRC patients was 6.1 months (*P* < 0.001). Moreover, the median OS of patients with single-organ pulmonary metastases was 41.1 months, while that of other mCRC patients was 18.7 months (*P* < 0.001) (Fig. 1).

Patients with prior BEV exposure had significantly shorter PFS than those without prior BEV exposure (mPFS: 5.6 months vs. 8.6 months,* P* = 0.0051). Median PFS in first line treatment was 9.5 months [0.8–68.5 months] and patients with long PFS in first line treatment (≥ 9.5 months) tended to have longer PFS in second line treatment (HR: 0.76, 95% CI: 0.55–1.04; *P* = 0.084) than those with short PFS in first line traetment (< 9.5 months).

Patients with *NRAS* Q61-mutated mCRC tended to have a longer PFS (*P* = 0.09) on univariate analysis. In the multivariate analysis, prior BEV exposure in first-line chemotherapy (hazard ratio [HR] 1.42, *P* = 0.016) and ECOG PS (≧1) (HR 1.57, *P* = 0.0088) were independent predictors of shorter PFS and single-organ pulmonary metastasis (HR 0.35, *P* = 0.00075) was an independent predictor of longer PFS.

Furthermore, patients with single-organ pulmonary metastasis had a significantly longer OS (HR 0.2, *P* = 0.006) compared to those with other forms of mCRC. ECOG PS (HR 1.75, *P* = 0.011) was also an independent predictor of shorter OS than others (Table 2). Grade 3 or 4 AEs were observed in 168 patients (58.1%) in the study cohort, with the most common AEs being neutropenia (49.1%), hypertension (10.0%), and proteinuria (8.0%). Grade 3 or 4 AEs occurred in 21 patients with single-organ pulmonary metastases and 147 patients with other forms of mCRC (80.8% vs. 55.9%, *P* = 0.02), and patients with single-organ pulmonary metastases experienced severe AEs more frequently than those without. The most common grade 3 or 4 AE was neutropenia (71.4%). In this study, in addition to BEV, we used AFL or RAM as a VEGF inhibitor and patients treated with these types of VEGF inhibitors had experienced AEs, such as neutropenia, hypertension, and proteinuria more frequently than those treated with BEV. This result was comparable with previous reports [28]. However, nearly all the events were manageable and few patients changed regimens because of intolerance.

**Table 2:** Cox proportional hazard analysis for progression-free survival (PFS) and overall survival (OS) in metastatic colorectal cancer patients treated with second-line chemotherapy

	**Univariate analysis**				**Multivariate analysis**			
**PFS**	**HR**	**Lower 95% CI**	**Upper 95% CI**	***P* value**	**HR**	**Lower 95% CI**	**Upper 95% CI**	***P* value**
Age (< 65^a^ or ≥ 65 years)	1.06	0.8	1.4	.69				
Sex (female^a^ or male)	0.81	0.62	1.08	.15				
ECOG PS (0 or 1, 2)	1.71	1.22	2.39	.0017	1.57	1.12	2.19	.0088
Primary tumor location (left^a^ or right)	1.32	0.98	1.78	.068				
Single organ pulmonary metastases (negative^a^ or positive)	0.32	0.17	0.58	.00023	0.35	0.19	0.64	.00075
Tissue *RAS* mutation (negative^a^ or positive)	0.98	0.74	1.31	.89				
Tissue *NRAS* Q61 mutation (negative^a^ or positive)	0.42	0.16	1.14	.09				
Prior bevacizumab exposure (in first-line chemotherapy; negative^a^ or positive)	1.5	1.13	1.99	.0055	1.42	1.07	1.89	.016
Treatment regimen (bevacizumab or others^a^)	1.1	0.83	1.46	.49				
	**Univariate analysis**				**Multivariate analysis**			
**OS**	**HR**	**Lower 95% CI**	**Upper 95% CI**	***P*** **value**	**HR**	**Lower 95% CI**	**Upper 95% CI**	***P*** **value**
Age (< 65^a^ or ≥ 65 years)	0.97	0.66	1.43	.89				
Sex (female^a^ or male)	1.01	0.68	1.48	.98				
ECOG PS (0 or 1, 2)	1.99	1.29	3.06	.0018	1.75	1.13	2.7	.011
Primary tumor location (left^a^ or right)	1.33	0.89	2.00	.16				
Single organ pulmonary metastases (negative^a^ or positive)	0.18	0.056	0.56	.0032	0.2	0.062	0.63	.006
Tissue *RAS* mutation (negative^a^ or positive)	1.21	0.81	1.82	.35				
Tissue *NRAS* Q61 mutation (negative^a^ or positive)	0.87	0.32	2.38	.78				
Prior bevacizumab exposure (in first-line chemotherapy; negative^a^ or positive)	1.46	0.98	2.17	.064				
Treatment regimen (bevacizumab or others^a^)	0.93	0.63	1.36	.69				

*CI* Confidence interval, *HR* Hazard ratio, *OS* Overall survival, *ECOG PS* Eastern Cooperative Oncology Group Performance status, *PFS* Progression-free survival, *RAS* Rat sarcoma viral oncogene homolog

^a^reference variables

## Discussion

This retrospective study demonstrated that patients with single-organ pulmonary metastases have a longer PFS and OS than those with other forms of mCRC in patients treated with FOLFIRI and VEGF inhibitors as second-line chemotherapy. The study findings might have an impact on therapeutic strategies for mCRC patients with single-organ pulmonary metastasis.

There could be several reasons for the better prognosis in mCRC patients with single-organ pulmonary metastases than those with other forms of mCRC as evidenced by our study. First, according to the biomarker findings, the total tumor volume was comparatively smaller than that for mCRC with other metastatic lesions. More specifically, in our study, the levels of tumor markers (CEA and CA19-9) were low at the initiation of second-line chemotherapy in patients with single-organ pulmonary metastases, which indicates that the total tumor volume is smaller in cases with single-organ pulmonary metastasis than in those with other forms of mCRC. The malignant grade in these types of tumors may also be low, and CEA and CA19-9 may not be secreted aggressively. Moreover, the mean LDH at the initiation of second-line chemotherapy tended to be lower in cases with single-organ pulmonary metastasis. Overall, these findings suggest that the total tumor volume might be low in patients with single-organ pulmonary metastasis.

The number of pulmonary lesions contributes to prognostic significance in CRC [29]. Furthermore, Miyake et al. showed that the tumor doubling time (TDT) of metastatic lesions in CRC was related to OS and that the TDT of pulmonary metastases was greater than that of liver metastases [30], which suggests that the OS of mCRC patients with single-organ pulmonary metastases was better than that of those without metastases due to a smaller tumor volume and a longer TDT.

In addition, there is a possibility that VEGF inhibitors are therapeutically effective in mCRC patients with single-organ pulmonary metastasis. A study by Ghouse et al. showed that therapeutic targeting of the vasculature in the premetastatic and metastatic niches reduced pulmonary metastases [21]. Shen et al. also revealed that tissue stiffness was higher in liver metastases than that in primary colorectal tumors. Highly activated metastasis-associated fibroblasts increase tissue stiffness, which enhances angiogenesis and anti-angiogenic therapy resistance [31]. Considering the good prognosis evidenced in our study, we hypothesized that if the metastasis-associated fibroblast activity and the tissue stiffness of pulmonary metastases are lower than that of liver metastases, anti-angiogenic therapy may be effective. Therefore, future studies must focus on modulating the mechanical microenvironment for therapeutic regimens. However, our study did not include a control group to enable this analysis. Therefore, additional studies are needed to verify our hypotheses.

It should also be considered that a good prognosis may also be related to genetic status. Several studies have uncovered a relationship between genetic mutations and pulmonary metastasis. Alamo et al. demonstrated higher pulmonary metastasis and poor survival rates with *KRAS* G12V mutations in colorectal cancer [32]. The mechanisms by which *KRAS* G12V mutation drives aggressive pulmonary tumor growth are not fully known. However, some studies have reported that *KRAS* G12V mutations upregulate the expression of CXCR4 and proinflammatory genes in the tumor microenvironment, leading to immune suppression and promoting tumor growth and metastases [33, 34].

This study could not identify the correlation between *KRAS* G12V mutations and pulmonary metastases. In our retrospective analysis, *NRAS* Q61-mutated mCRC cases with single-organ pulmonary metastases showed a tendency towards a longer PFS (*P* = 0.09). Giannou et al. showed that *NRAS* mutations promote colonization of the lungs by various tumor types in mouse models [35]. However, Ikoma et al. investigated the prognostic features of patients with *RAS* mutant CRC in Japan and showed that *NRAS* mutant CRC tended to have a short OS [36]. The number of *NRAS*-mutated CRC cases was small in our cohort, and our findings require replication in larger investigations. Moreover, in our study, there were no BRAF V600E-mutated cases of single-organ pulmonary metastases.

In general, BRAF mutations may confer mCRC with a worse prognosis as well as resistance to chemotherapy [37]. The association between pulmonary metastases and BRAF V600E mutations has not been studied. However, these genetic factors might have a good prognosis in cases with single-organ pulmonary metastases arising from mCRC. Our results showed a relationship between distinctive genetic status and good prognosis in mCRC patients with single-organ pulmonary metastasis. However, further comprehensive analysis is needed to validate this relationship.

Based on the results of our retrospective study, we suggest several therapeutic strategies for mCRC with single-organ pulmonary metastases. First, our study revealed that the prognosis for single-organ pulmonary metastasis in mCRC patients treated with FOLFIRI and VEGF inhibitors was generally favorable. Therefore, these patients may not necessarily require intensive chemotherapy for mCRC. In this retrospective study, we assumed that the patients with single-organ pulmonary metastases have a good prognosis and are a less aggressive subgroup considering their biological behavior. However, the better prognosis could be attributed to the therapeutic agents. Therefore, prospective clinical trials are needed to determine whether non-intensive treatment is acceptable for these patients. In addition, as the prevalence of mCRC is increasing, the number of older patients and those with multiple comorbidities undergoing chemotherapy has also increased [38]. In view of this increase, we propose that reduced-intensity chemotherapy may be implemented for mCRC patients with single-organ pulmonary metastases. If these strategies are introduced, many unnecessary AEs can be avoided and good performance status (PS) can be maintained in patients, thereby improving their treatment efficacy and quality of life.

Next, regarding treatment strategies (including resection of metastases), previous studies have reported that pulmonary resection for metastases arising from CRC may contribute to prolonged OS [39]. Our study reports a good prognosis for single-organ pulmonary metastases following second-line chemotherapy, even though the RR for second-line chemotherapy for mCRC is approximately 10–20% (which is lower than that for first-line chemotherapy) [40] and has the possibility of suppressing tumor growth for a long time in mCRC patients with single-organ pulmonary metastases. However, some of these patients may be eligible for surgery. Despite the difficulty in performing curative surgery, a potential strategy that combines surgery with several locoregional therapies, such as SRT and RFA, could be implemented for successful R0 resection.

This study might have an impact on therapeutic strategies for synchronous liver and pulmonary metastases. Mise et al. conducted a retrospective analysis showing that patients with liver and unresectable, low-volume pulmonary metastases arising from CRC demonstrated improved survival with liver resection as compared to chemotherapy alone [41]. In addition, another study demonstrated a relationship between the site of metastasis and the cause of death and reported that liver metastases are the most common cause of death in mCRC patients [42]. Therefore, in mCRC patients with both liver and lung metastases, R0 resection of liver metastases may lead to prolonged survival. Currently, a prospective, randomized trial of liver resection vs. no surgery in patients with liver and unresectable pulmonary metastases arising from CRC is being carried out to support the findings of a previous retrospective study conducted by the same group [41]. Based on the results of this randomized study, clinicians might need to plan a therapeutic strategy of chemotherapy for unresectable pulmonary metastases following debulking surgery for liver metastases.

Despite the major strengths of our study as elucidated, we also acknowledge several limitations of our work herein. First, the retrospective nature of our study is the major limitation of this work as this precludes treatment assignments and drawing causal inferences. In addition, this study included a small sample size, limiting the study’s statistical power. Finally, the study did not include control cases that were treated with FOLFIRI alone, limiting the generalizability of its findings.

## Conclusions

In conclusion, the study showed that the prognosis of mCRC patients with single-organ pulmonary metastases treated with FOLFIRI and VEGF inhibitors as second-line chemotherapy was significantly better than that of patients with other forms of mCRC. In the future, if larger prospective studies confirm these findings, novel therapeutic strategies may be available for these patients.

## Data Availability

The datasets generated during and/or analyzed during the current study are not publicly available due but are available from the corresponding author upon reasonable request.

## Abbreviations

5-FU: 5-Fluorouracil
AEs: Adverse events
AFL: Aflibercept
BEV: Bevacizumab
CA19-9: Carbohydrate antigen 19–9
CEA: Carcinoembryonic antigen
CRC: Colorectal cancer
CR: Complete response
CTCAE: Common Toxicity Criteria for Adverse Events
DCR: Disease control rate
FOLFIRI: Leucovorin + fluorouracil + irinotecan
JSCCR: Japanese Society for Cancer of the Colon and Rectum
LDH: Lactate dehydrogenase
mCRC: Metastatic colorectal cancer
OS: Overall survival
PD: Progressive disease
PFS: Progression-free survival
PR: Partial response
PS: Performance status
RAM: Ramucirumab
RFA: Radiofrequency ablation
RR: Response rate
SD: Stable disease
SRT: Stereotactic radiation therapy
TDT: Tumor doubling date
VEGF: Vascular endothelial growth factor
